# Use of potentially inappropriate medications and adverse events in older outpatients with acute conditions

**DOI:** 10.31744/einstein_journal/2022AO8024

**Published:** 2022-06-28

**Authors:** Stéphanie de Souza Costa Viana, Natália Pereira dos Santos Souza, Márlon Juliano Romero Aliberti, Wilson Jacob-Filho

**Affiliations:** 1 Hospital das Clínicas Faculdade de Medicina Universidade de São Paulo São Paulo SP Brazil Hospital das Clínicas, Faculdade de Medicina, Universidade de São Paulo, São Paulo, SP, Brazil.; 2 Escola de Artes, Ciências e Humanidades Universidade de São Paulo São Paulo SP Brazil Escola de Artes, Ciências e Humanidades, Universidade de São Paulo, São Paulo, SP, Brazil.

**Keywords:** Drug-related side effects and adverse reactions, Frail elderly, Aged, Potentially inappropriate medication list

## Abstract

**Objective:**

To examine associations between potentially inappropriate medication, use and the risk of falls, unplanned hospitalization and death in older patients receiving initial care in a geriatric day hospital due to acute conditions.

**Methods:**

Cohort study with older adults referred to a geriatric day hospital from 2014 to 2017 due to acute conditions. Patients were submitted to comprehensive geriatric assessment. Use of medications was analyzed according to Beers Criteria 2019. Outcome assessment was based on monthly follow-up telephone calls made over the course of one year.

**Results:**

In this sample, 40.6% of patients had been prescribed at least one potentially inappropriate medication, particularly proton pump inhibitors (66.5%). Over the course of follow-up, 44.7% of patients receiving potentially inappropriate medications sustained at least one fall (p=0.0043) and 70% visited the emergency department (p=0.0452). These outcomes were more common among patients using two or more of drugs. Use of potentially inappropriate medication was associated with a 64% increase in the odds of unplanned hospitalization and a two-fold increase in risk of death.

**Conclusion:**

Associations between potentially inappropriate medication use and unfavorable outcomes such as falls and unplanned hospitalizations within one year of admission to a geriatric day hospital support the application of Beers Criteria and emphasize the importance of periodic prescription review, deprescription and rational use of these drugs whenever possible.

## INTRODUCTION

The prescription of medications is a fundamental component of older patient care and is often a complex and challenging task, which is non-generalizable due to the particularities of care in this population. Medication prescription is associated with the occurrence of adverse events, higher rates of hospital admission, higher morbidity and mortality in older adults when not appropriately monitored. Atypical adverse drug reactions in this age group makes their recognition difficult.^(1-5)^

Several methods and instruments have been developed to evaluate prescription quality and detect potentially inappropriate medication (PIM) prescription, in an effort to minimize risks of inappropriate medication use in older adults. Of these, Beers Criteria are the best known and most widely used worldwide.^(6-8)^

Inappropriate prescription is thought to be a common and preventable occurrence, which has been associated with falls and unplanned hospitalizations, among other problems. Detection of inappropriate prescription may reduce the risk of falls and related adverse events.^(6,9-11)^

Assessment of the applicability of these findings to the Brazilian population is a crucial aspect of care in older patients in order to determine and guide care provision and drug prescription, given the peculiarities of this population.

## OBJECTIVE

To examine associations between potentially inappropriate medication, use and the risk of falls, unplanned hospitalization and death in older patients receiving initial care in a geriatric day hospital due to acute conditions.

## METHODS

### Study population and design

This cohort study was carried out between 2014 and 2017 with older patients seen at a geriatric day hospital aimed to provide short-term care for patients with acute or acute chronic diseases potentially amenable to hospitalization. Additional information can be obtained in a prior study published by Aliberti et al.^(12)^

### Participants

Patients aged 60 years or older referred to a geriatric day hospital due to acute conditions or exacerbation of chronic diseases were evaluated by a multi-professional team. Of these, patients receiving exclusively palliative care or presenting with clinical emergencies requiring immediate hospitalization were excluded. Other exclusion criteria were refusal to participate (patient) or of consent to participate (legal representative when applicable), lack of reliable telephone contact for follow-up and outcome measurement, elective hospitalization scheduled within the next 12 months; those who did not use drugs continuously and inability to walk.

### Data collection

Upon admission, patients were submitted to a comprehensive geriatric assessment lasting about 50 minutes for data collection. The following data were collected: sociodemographic data (age, sex, race/ethnicity, years of formal education and monthly household income *per capita*), caregiver support, multimorbidities (Charlson Comorbidity Index),^(13)^ continuous use of medicines (>3 months; self-reported or obtained via electronic prescription system), Pharmacotherapy Complexity Index (PCI),^(14)^ cognitive function (10-point cognitive screener, 10-CS),^(15)^ symptoms of depression (Geriatric Depression Scale, GDS-4)^(16)^ and frailty defined according to Fried’s five frailty phenotype criteria and measures used in the Cardiovascular Health Study.^(17)^

### Potentially inappropriate medication

Beers Criteria 2019 (second table listing drugs to be avoided in all or most older adults)^(6)^ compared with medical prescriptions available in the hospital electronic system and/or presented during the interview were used for identification of PIM. Data extracted from medical records were also used to check indications for prescription of certain drugs in cases in which exceptions for use were described.

### Follow-up

Patients or caregivers received monthly telephone calls for 12 months. Phone calls were made by a member of the research team blinded to data collected at the initial evaluation. Follow-up data were collected using a standardized questionnaire administered to patients or caregivers, as appropriate. Questions were related to falls, visits to the emergency department, hospitalizations and death.

### Ethical issues and financing

This study is part of a larger study named “Day - Hospital for the elderly with risk of hospitalization” (*Hospital Dia para idosos com risco de hospitalização*), approved by the Ethics Committee for Research Projects Analysis, (CAAE: 37322214.1.0000.0068, # 898.108) and financed by *Programa de Pesquisa para o Sistema Único de Saúde* (PPSUS) - 2014, a partnership between the Ministry of Health and *Fundação de Amparo à Pesquisa do Estado de São Paulo* (FAPESP), from July 2014 to December 2015 (# 2014/50007-4). This was the implementation of the consent form to data use for future studies, eliminating the need to sign a new informed consent form for the study in question.

This study was submitted to and approved by the Ethics Committee for Research Projects Analysis of *Hospital das Clínicas, Faculdade de Medicina, Universidade de São Paulo* (HCFMUSP) São Paulo (SP), Brazil, (CAAE: 60888016.4.0000.0068; #1.790.023) and supported by the *Coordenação de Aperfeiçoamento de Pessoal de Nível Superior* (CAPES), Financing code 001.

### Statistical analyses

Categorical variables were expressed as numbers and percentages and compared using the χ^2^ test. Continuous variables were expressed as measures of central tendency (mean or median) and dispersion (standard deviation, SD) or interquartile ranges (IIQ), according to the distribution of the sample. Continuous variables with normal distribution were compared using the Student *t-*test for independent samples. Whenever non-normal distribution was identified, comparisons were made using the Wilcoxon-Mann-Whitney or the Kruskal Wallis test in the case of more than one category.

Cumulative incidence curves were constructed to illustrate the occurrence of adverse outcomes according to use of PIM (none, 1 PIM or 2 or more PIMs). Associations between use of PIM and adverse outcomes were examined using the Fine-Gray model, in which death was considered a competing event. Univariate and multivariate models also included sociodemographic factors, Charlson comorbidity index, number of medications used, 10-CS, GDS-4 and frailty status. Sub-hazard ratios (sub-HR) and 95% confidence intervals (95%CI) were provided for the variable of primary interest (use of PIM). The same models were fitted to the outcome mortality using the Cox proportional hazard regressions.

Analyses were rerun using inverse probability weighting (IPW) to account for the probability of each patient receiving PIM. Inverse probability weighting is a technique used to eliminate confounding by indication via adjustment for reasons that influence clinician’s decision making regarding a specific treatment. In this study, IPW was computed using a logistic regression model in which PIM was the outcome and sociodemographic and clinical measures (age, sex, race/ethnicity, income, education, Charlson comorbidity index, cognition, depressive symptoms, pharmacotherapy complexity index and frailty) were predictors.

All statistical tests were two-tailed, with a level of significance <0.05. Analyses were performed using software (Stata 14; StataCorp, College Station, TX).

## RESULTS

Between 2014 and 2017, 1,067 patients admitted to a geriatric day hospital were eligible to participate in the study. Patients receiving exclusively palliative care (n=41) and patients who were unable to walk (n=39), required full-time hospital care (n=74), were not taking any drugs (n=20) or refused to participate (n=25) were excluded. The final sample comprised 868 patients.

Participants were aged 79.2 (±8.2) years, with the following age range distribution: 60 and 79 years, 48.3%; 80 years or more, 51.7%. Most participants were females (64.2%) with white skin color (59.7%), 4 years of formal education on average and caregiver support (55.6%). Regarding frailty, 36.5% of patients were considered pre-frail and 56.1% frail (Fried’s five phenotype criteria), with increased risks of adverse events associated with medication use (Table 1).

**Table 1:** Characteristics of older adults seen at a geriatric day hospital

Variable	
Age, mean (SD)	79.2±8.2
Sex, n (%)	
Male	311 (35.8)
Female	557 (64.2)
Race/ethnicity, n (%)	
White	518 (59.7)
Non-white	350 (40.3)
Monthly household income per capita, n (%)	
≤1 minimum wage	490 (56.4)
Education ≤4 years, n (%)	535 (61.6)
Caregiver, n (%)	483 (55.6)
Charlson Comorbidity Index, n (%)	
0	120 (13.8)
1-2	367 (42.2)
3 or more	381 (44.0)
Number of drugs used, median (IIQ)	8.5 (6-11)
Pharmacotherapy complexity index, median (IIQ)	26.5 (17.5-36)
10-point Cognitive Screener	
Probable dementia (0-5)	314 (36.2)
Possible dementia (6-7)	194 (22.3)
Normal cognition (8-10)	360 (41.5)
Symptoms of depression (GDS-4)	
0	104 (12.0)
1-2	594 (68.4)
3-4	170 (19.6)
Frailty, n (%)	
Robust	64 (7.4)
Pre-frail	317 (36.5)
Frail	487 (56.1)

SD: standard deviation; IIQ: interquartile range; GDS-4: Geriatric Depression Scale.

Major reasons for referral to a geriatric day hospital were decompensated *diabetes mellitus* (16.8%), acute anemia (15.6%) and heart failure (functional class III or IV according to the New York Heart Association: https://professional.heart.org/en/guidelines-and-statements/classification) (12.6%).

A median of 8.5 medications used per individual (95%CI: 6-11) was recorded upon admission. Prescription of PIM was detected in 40.6% of cases. Proton pump inhibitors (PPIs) were the most commonly prescribed class of PIM (66.5%). The maximum number of PIMs per individual was 4 (Table 2). Prescription of antidepressants, muscle relaxants and benzodiazepines was detected in 12.2%, 10.5% and 8.8% of cases respectively (Table 3).

**Table 2:** Distribution according to number of potentially inappropriate medications used by patients

Number of PIM use	n (%)
None	516 (59.4)
One PIM	281 (32.4)
Two PIMs	53 (6.1)
Three or more PIMs	18 (2.1)

PIM: potentially inappropriate medication.

**Table 3:** Pharmacological class and potentially inappropriate medications most commonly prescribed for older adults

Pharmacological class (PIM) PIM most commonly prescribed	n (%)
PPI	234 (66.5)
Omeprazole	234 (100)
Antidepressants	43 (12.2)
Paroxetine	22 (51.2)
Amitriptyline	13 (30.2)
Muscle relaxants	37 (10.5)
Carisoprodol	31 (83.8)
Cyclobenzaprine	6 (16.2)
Benzodiazepines	31 (8.8)
Clonazepam	14 (45.2)
Bromazepam	7 (22.6)

PIM: potentially inappropriate medication; PPI: proton pump inhibitors.

Comparisons between PIM and non-PIM drug classes revealed associations between the Pharmacotherapy Complexity Index^(14)^ and the use of PIMs (median 31.8 [IIQ 21.0-42.6]) or non-PIMs (median 25 [IIQ 16.9-35.0]) (p<0.001), with stronger associations in the subgroup of interest.

In this cohort, 45.9% of participants reported at least one fall during the follow-up year period. Of these, 44.7% used at least one PIM. In this sample, approximately 70% of older patients sought emergency care, for different reasons. Of these, 67.7% and 76.7% used 1 PIM or 2 or more PIMs respectively. Unplanned hospitalization during the follow-up period was detected in 37.2% of the sample. Of hospitalized patients, 39.1% were using one PIM and 38.4% two or more PIMs, with no associations between variables of interest.

**Figure 1:**
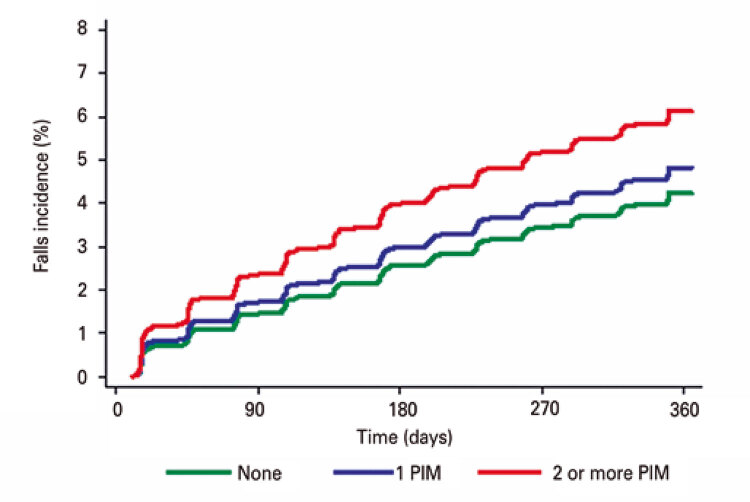
Incidence curves of falls over the course of one year according to the number of potentially inappropriate medications prescribed for older patients treated at the geriatric day hospital

**Figure 2:**
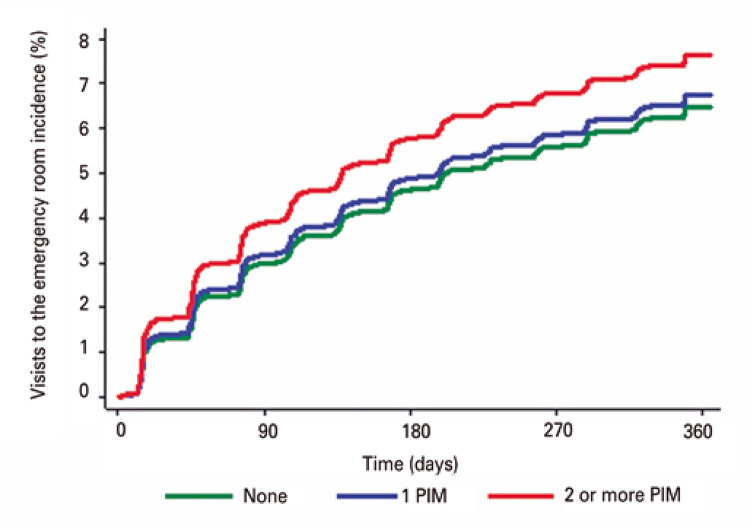
Incidence curves of visits to the emergency department over the course of one year according to the number of potentially inappropriate medications prescribed for older patients at the geriatric day hospital

**Figure 3:**
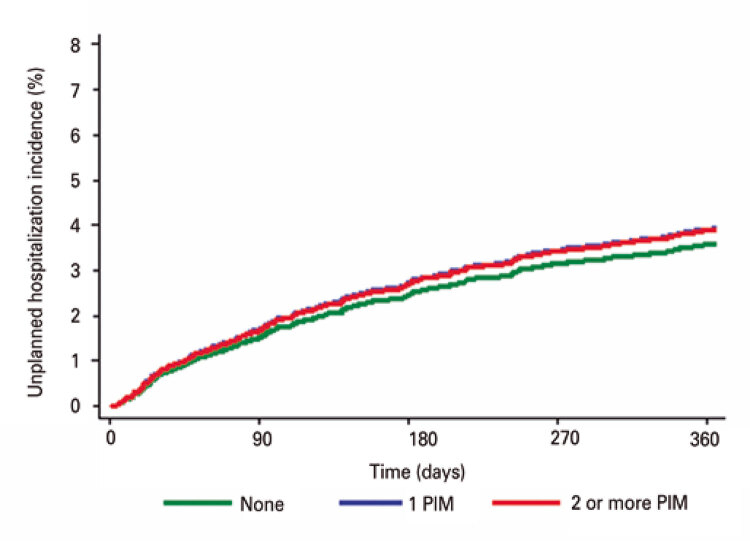
Incidence curves of unplanned hospitalization over the course of one year according to the number of potentially inappropriate medications prescribed for older patients at the geriatric day hospital

**Figure 4:**
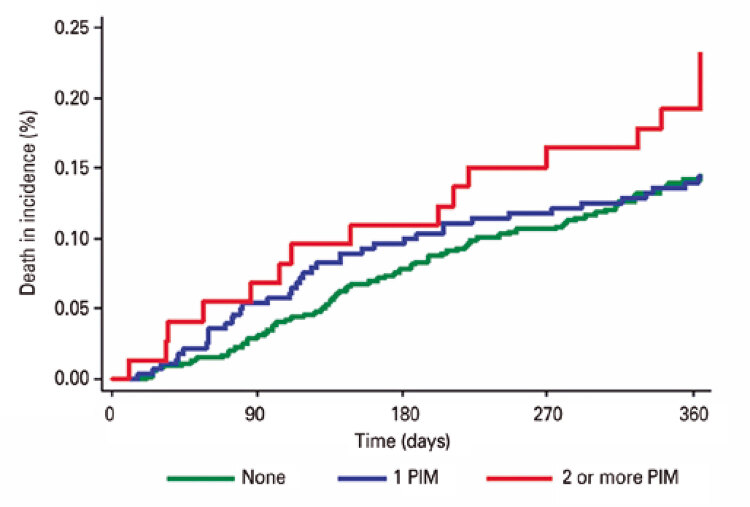
Incidence curves of death over the course of one year according to the number of potentially inappropriate medications prescribed for older patients at the geriatric day hospital

Over the course of the 1-year follow-up, the cumulative incidences of falls (Figure 1), visits to the emergency department (Figure 2), hospitalizations (Figure 3) and death (Figure 4) were similar between patients taking no PIMs or only one PIM. However, a higher incidence of falls, visits to the emergency department and death was detected in patients taking two or more PIMs relative to those not taking PIMs. Hospital admissions were not associated with use of PIM.

Detailed analysis of patients who used PIMs and sustained falls, visited the emergency department or died revealed omeprazole was the most commonly prescribed PIM.

Patients taking two or more PIMs had a higher risk of falling, visiting the emergency department and dying over the course of the one-year follow-up. Results remained consistent after application of the IPW technique (*i.e*., higher risk of fall, visit to the emergency department and mortality among patients using two or more PIMs). Of note, after application of IPW patients using two or more PIMs had a higher risk of hospital admission relative to those not taking PIMs (Table 4).

**Table 4:** Associations between use of potentially inappropriate medication and adverse outcomes within one year of admission to the geriatric day hospital

Outcomes/PIM use	Sub-hazard ratio or hazard ratio (95% confidence interval)			
	Prior to IPW		After IPW	
	Univariate	Adjusted*	Univariate	Adjusted*
Fall				
No PIM	(reference)	(reference)	(reference)	(reference)
1 PIM	1.20 (0.97-1.48)	1.19 (0.94-1.50)	1.16 (0.94-1.45)	1.27 (1.00-1.61)
≥2 PIMs	1.72 (1.24-2.40)	1.66 (1.17-2.35)	1.61 (1.09-2.38)	1.86 (1.24-2.80)
Hospitalization				
No PIM	(reference)	(reference)	(reference)	(reference)
1 PIM	1.12 (0.89-1.42)	1.11 (0.87-1.44)	1.11 (0.87-1.41)	1.10 (0.85-1.43)
≥2 PIMs	1.11 (0.74-1.67)	1.18 (0.77-1.80)	1.54 (1.02-2.31)	1.64 (1.11-2.44)
Visit to the ED				
No PIM	(reference)	(reference)	(reference)	(reference)
1 PIM	1.08 (0.90-1.28)	1.01 (0.84-1.22)	1.03 (0.85-1.24)	1.03 (0.84-1.26)
≥2 PIMs	1.38 (1.05-1.83)	1.38 (1.03-1.85)	1.56 (1.17-2.07)	1.70 (1.27-2.28)
Mortality				
No PIM	(reference)	(reference)	(reference)	(reference)
1 PIM	1.01 (0.69-1.49)	1.05 (0.70-1.60)	1.01 (0.68-1.50)	1.06 (0.69-1.60)
≥2 PIMs	1.59 (0.93-2.73)	2.04 (1.14-3.63)	2.11 (1.15-3.87)	2.19 (1.23-3.92)

* Adjusted for sociodemographic factors (age, sex, race/ethnicity, education, income), Charlson comorbidity index, number of medications used, 10-point Cognitive Screener, 4-item Geriatric Depression Scale and frailty status. Outcomes fall, hospitalization and visit to the emergency department were analyzed using the Fine-Gray method, in which death was considered a competing event. Mortality was analyzed using Cox proportional hazard models.

IPW: inverse probability weighting; PIM: potentially inappropriate medication; ED: emergency department.

Outcomes fall, hospitalization and visit to the ED were analyzed using the Fine-Gray method, in which death was considered a competing event. Mortality was analyzed using Cox proportional hazard models.

## DISCUSSION

Telephone follow-up revealed associations between PIM use, falls and visits to the emergency department within one year of admission to a GDH. These outcomes, as well as unplanned hospitalizations and death, were more likely among patients who used two or more potentially inappropriate medications.

In this sample, polypharmacy reflected the complexity of the population studied and increased the risk of adverse events.^(2-4)^ The number of drugs used was associated with PIM prescription, regardless of the context of care. The higher the number of drugs prescribed, the higher the chances of PIM use.^(18)^

Associations between use of PIMs and complexity of pharmacotherapy detected in this study (<0.001), reflect a population in need of a high number of medicines due to comorbidities. Detailed prescriptions and appropriate monitoring are needed to promote correct use of medications and facilitate treatment adherence.^(14)^

Data extracted from medical prescriptions of participants at the time of admission indicated that, out of 868 patients in this cohort, approximately 40% were using at least one PIM, PPIs being the most common (66.5%), particularly omeprazole.

Use of PPIs has increased worldwide, including inappropriate use. In a study conducted in Rio de Janeiro (RJ), Brazil, 29.5% of the sample reported the use of omeprazole with no clinical indication described in medical records. In this study, duration of use increased with age.^(19,20)^

The inclusion of PPIs in the list of Beers Criteria was a milestone in health care. Since PPIs are thought to be safe and well-tolerated, they are often prescribed with no clear indications and prescriptions maintained for years, without proper evaluation. These drugs may be potentially inappropriate for older adults due to increased risks of falls, fractures, and intestinal infections by *Clostridium difficile*.^(6)^

Falls and related consequences are major public health concerns, which often require medical intervention and may be associated with medication use. In a retrospective cohort study carried out by Bor et al. polypharmacy was associated with higher rates of falls (p=0.010) in older patients. In this study, use of PPI, particularly pantoprazole, was associated with a 2.5-fold increase in the risk of falling.^(21,22)^ Likewise, use of long-acting benzodiazepines (7.8%) and drugs with anticholinergic properties (5.0%) were associated with falls in a study performed by Berdot et al.^(23)^ These findings support data derived from this cohort.

Around 2 million people use muscle relaxants each year. Of these, approximately 300 thousand are aged 60 years or more.^(24)^ Muscle relaxants containing orphenadrine ranked first among top selling drugs in Brazil from 2013 to 2014.^(25)^

In a follow-up study by Billups et al.^(24)^ carisoprodol was one of the most commonly used muscle relaxants. Besides questionable effectiveness, these drugs are thought to be potentially inappropriate due to low tolerance, excessive sedation and resultant risk of falls and fractures in the older adults.^(6)^ Abuse of this drug is due to its metabolite meprobamate, which is responsible for tolerance effects and dependence.^(26)^

Benzodiazepines are often indicated to control insomnia and anxiety in older adults. These drugs are prescribed to 8-12% of the geriatric population in the United States, New Zealand and Germany, and to approximately 7% of older adults in Brazil. A prevalence of 9% of benzodiazepine use has been reported.^(27)^ In spite of the apparent declining effectiveness after the first 4 weeks of use due to tolerance development and the adverse effects of maintenance regimens, use of these medications is often sustained for long periods, leading to other health problems, such as drug dependence.^(6,27,28)^ These drugs are also are thought to be potentially inappropriate for older adults due to increased risk of fractures, cognitive impairment and delirium, and to elevated health care costs.^(6)^

This study revealed borderline associations between visits to the emergency department and PIM use (70% of patients). These associations were stronger in patients using two or more PIMs, as shown by incidence curves derived from Fine & Gray model analysis.

Approximately 30% of visits to the emergency department due to drug-related adverse events in the United States between 2013 and 2014 involved older adults (95%CI: 30.3-38.8), with 43.6% of patients requiring hospitalization. Use of potentially inappropriate drugs were implicated in 3.4% of cases.^(29)^

In this cohort, analyzes carried out using the IPW technique revealed associations between use of PIM and visits to the emergency department, with increased odds of 38% (univariate analysis) and 70% (multivariate analysis). These findings emphasize the significance of careful drug use in older adults.

Unplanned hospitalizations within one year were not associated with PIM prescription detected upon geriatric day hospital admission, with small differences in incidence between patients who used PIMs and those who did not. This finding may be explained by the complexity of patients in this cohort, which had a significant impact on this outcome. In spite of the low representativeness of the Charlson Comorbidity Index in this sample, 56.1% of patients were classified as frail and 36.5% as pre-frail, implying greater vulnerability and higher risks of clinical instability and decompensation requiring hospitalization.^(30)^

Identification of PIM may be a valid strategy to improve pharmacotherapy among older adults, since it may guide prescription review and promote deprescription. These strategies are vital for patient-centered care in older adults with polypharmacy, frailty or questionable benefits of therapy (*e.g*., patients with advanced dementia).^(31)^ Another important point is the need to educate health professionals involved in care of older patients in order to increase awareness of the risks of PIM use. Part of this training might involve the use of computerized systems that can easily identify PIM and help health care providers to reduce their use.^(31)^ Periodic review of medications used should be performed, including potential variations, adherence to treatment, indications for use and time to benefit of each medication. Non-pharmacological therapies should be adopted whenever possible.^(30)^

## CONCLUSION

Associations between potentially inappropriate medications and unfavorable outcomes in Brazilian outpatients seen at a high-complexity geriatric day hospital supports the application of Beers Criteria, particularly the current versions, as a guideline for safe and rational use of medicines. Findings of this study emphasize the importance of reviewing strategies, frequent deprescription and monitoring of medication use in order to minimize the chances of adverse events in this population.
